# Long-Term Trends and Determinants of Tuberculosis Burden in China, 1990–2023: Insights from the Global Burden of Disease Study 2023

**DOI:** 10.3390/pathogens15030295

**Published:** 2026-03-08

**Authors:** Yingxing Wang, Guozhong He, Hoiman Ng, Chaoxi Niu, Rong Li, Furong Zhang, Ruimei Shi, Xingyue Dian, Qingping Ma, Zhong Sun

**Affiliations:** 1 School of Public Health, Kunming Medical University, Kunming 650500, China20241972@kmmu.edu.cn (R.L.); 20230256@kmmu.edu.cn (F.Z.); 20250276@kmmu.edu.cn (R.S.);; 2 Clinical Laboratory, Kiang Wu Hospital, Macau 999078, China; alvin_ng@163.com; 3 Faculty of Data Science, City University of Macau, Macau 999078, China; cxniu@cityu.edu.mo

**Keywords:** tuberculosis, China, Global Burden of Disease, age–period–cohort analysis, demographic transition, risk factors, latent tuberculosis infection, population aging

## Abstract

Tuberculosis (TB) remains a major public health challenge in China despite substantial long-term progress. Using data from the Global Burden of Disease Study 2023, this study reassessed trends and determinants of TB burden in China from 1990 to 2023. Age-standardized incidence, mortality, and disability-adjusted life year (DALY) rates were analyzed using estimated annual percentage change, age–period–cohort modeling, and demographic decomposition, with comparative risk assessment to quantify behavioral and metabolic contributions. Between 1990 and 2023, age-standardized incidence, mortality, and DALY rates declined by approximately 73.24%, 94.00%, and 92.40%, respectively. Negative net and local drift values indicated sustained reductions across age groups; however, the decline slowed after 2021, with a modest rebound in incidence. Since 2015, reductions in incidence have been more moderate than the pace required to achieve the 2035 End TB Strategy targets. Decomposition analysis demonstrated that improvements in age-specific rates were the primary drivers of long-term reductions, whereas demographic shifts—particularly population aging—partially offset these gains. The burden increasingly shifted toward older adults, and males consistently experienced higher rates than females. Tobacco and alcohol use contributed substantially to sex differentials, while undernutrition and metabolic disorders remained relevant risk factors. These findings indicate that China’s TB epidemic has entered a phase shaped by demographic aging and evolving risk structures, requiring sustained and adaptive control efforts.

## 1. Introduction

Tuberculosis (TB), caused by *Mycobacterium tuberculosis*, remains one of the most persistent and deadly infectious diseases worldwide. An estimated one-quarter of the global population carries latent tuberculosis infection (LTBI), representing a vast reservoir for future reactivation and sustained transmission [1]. According to the World Health Organization (WHO) Global Tuberculosis Report 2024, approximately 10.6 million individuals developed TB and 1.25 million died from the disease in 2023, making TB the leading cause of death from a single infectious agent globally [2]. In response to this enduring burden, the WHO End TB Strategy established ambitious milestones: a 90% reduction in TB incidence and a 95% reduction in TB mortality by 2035 compared with 2015 levels, with TB elimination—defined as fewer than one case per million population annually—targeted by 2050 [3]. Although global TB incidence has declined by approximately 19% since 2015, the current pace of reduction remains insufficient to meet these milestones, particularly in high-burden settings facing demographic transitions, health system disruptions, and persistent risk factors such as diabetes and socioeconomic inequality [2,4,5]. This gap between strategic targets and epidemiological reality underscores the need for sustained surveillance, strengthened health systems, and adaptive, risk-informed TB control strategies.

China remains one of the countries bearing the highest absolute TB burden globally, consistently ranking among the top three nations in total incident cases since 2015 [2,6]. In 2023, approximately 741,000 new TB cases were reported in China, accounting for 6.8% of the global total [2]. Previous Global Burden of Disease Study (GBD)-based analyses covering 1990–2021 documented substantial and sustained reductions in TB incidence and mortality in China, with annual percentage declines of approximately 4–5% in age-standardized incidence rates [7,8]. Despite these achievements, the epidemiological landscape remains complex. Rapid population aging, persistent socioeconomic disparities, and the continued burden of multidrug-resistant tuberculosis (MDR-TB) pose structural challenges to sustained progress [6,7,8]. Given the End TB Strategy target of a 90% reduction in TB incidence by 2035 compared with 2015 levels, it remains uncertain whether the historical pace of decline will be sufficient to achieve this milestone under evolving demographic and epidemiological conditions.

As tuberculosis transmission declines, the relative contribution of modifiable behavioral and metabolic risk factors to disease burden has become increasingly prominent. Strong evidence indicates that behavioral factors such as smoking and alcohol consumption substantially increase the risk of tuberculosis infection, progression, and mortality, while metabolic abnormalities—including dysglycemia and abnormal body weight—further modify susceptibility and clinical outcomes [9,10,11,12,13]. In rapidly aging populations undergoing epidemiological transition, the convergence of chronic non-communicable diseases and tuberculosis has become increasingly evident, reflecting a growing bidirectional interaction between metabolic disorders and TB risk [14,15,16]. In China, where tobacco use remains highly prevalent among men and the burden attributable to metabolic risk factors has increased substantially over the past decades, there is growing recognition of the need to integrate tuberculosis control within broader chronic disease prevention frameworks [17,18,19]. A comprehensive reassessment of long-term TB trends alongside evolving risk structures is therefore essential to inform more precise and risk-targeted public health interventions.

Despite substantial progress documented in previous GBD-based analyses, most prior studies examining tuberculosis burden in China were limited to data up to 2021 and did not fully capture the epidemiological impact of the COVID-19 pandemic or subsequent recovery period [6,20]. Furthermore, earlier investigations often focused primarily on temporal trends without simultaneously integrating demographic decomposition and risk-attribution analyses within a unified framework. The release of the Global Burden of Disease Study 2023 provides the first opportunity to incorporate complete post-pandemic estimates and to reassess long-term tuberculosis patterns in the context of evolving demographic structures and modifiable risk factors. Therefore, this study aims to comprehensively evaluate trends in tuberculosis incidence, mortality, and disability-adjusted life years in China from 1990 to 2023, and to further quantify the contributions of demographic change and key behavioral and metabolic risk factors. By integrating trend analysis, age–period–cohort modeling, and risk attribution, this study seeks to generate updated evidence to inform precision-oriented tuberculosis control strategies.

## 2. Materials and Methods

### 2.1. Data Sources

Data for this study were obtained from the Global Burden of Disease Study 2023 (GBD 2023), conducted by the Institute for Health Metrics and Evaluation (IHME), University of Washington. GBD 2023 provides standardized annual estimates of incidence, mortality, disability-adjusted life years (DALYs), and age-standardized rates for 204 countries and territories from 1990 to 2023. For China, national-level estimates of tuberculosis incidence, mortality, DALYs, and corresponding age-standardized incidence rate (ASIR), age-standardized mortality rate (ASMR), and age-standardized DALY rate (ASDR) were extracted for the period 1990–2023, stratified by sex and five-year age groups, from the publicly available GBD Results Tool (https://vizhub.healthdata.org/gbd-results/ (accessed on 1 January 2026)). Estimates were generated using standardized modeling approaches, including the Cause of Death Ensemble model (CODEm) for mortality and the Bayesian meta-regression tool DisMod-MR 2.1 for non-fatal outcomes, ensuring internal consistency across age, sex, location, and time. Uncertainty intervals (95% UIs) were derived from 1000 posterior draws to account for sampling variability and model uncertainty.

### 2.2. Trend Analysis

Temporal trends in tuberculosis incidence, mortality, and DALYs were evaluated using age-standardized rates (ASRs) based on the GBD standard world population to account for changes in age structure over time. The magnitude and direction of long-term trends were quantified using the estimated annual percentage change (EAPC), calculated by fitting a log-linear regression model to ASRs over calendar years (ln[ASR] = α + β × year), with EAPC derived as 100 × (exp(β) − 1). An EAPC with a 95% confidence interval (CI) entirely above zero was considered indicative of an increasing trend, whereas a value entirely below zero indicated a decreasing trend; otherwise, the trend was considered stable. To explore potential shifts associated with the COVID-19 pandemic, segmented analyses were conducted for the periods 1990–2021 and 2021–2023. All statistical analyses were performed using R software (version 4.3.3).

### 2.3. Age–Period–Cohort (APC) Analysis

An APC model was applied to disentangle the independent effects of age, calendar period, and birth cohort on temporal trends in tuberculosis incidence, mortality, and DALYs. A log-linear Poisson regression framework was used to model age-specific rates across successive five-year age groups and calendar periods, with corresponding birth cohorts derived from the cross-classification of age and period intervals. To address the inherent identifiability problem among age, period, and cohort effects, the Intrinsic Estimator (IE) method was employed, which has been widely used in epidemiological trend analyses [4,21,22]. Rate ratios (RRs) and 95% confidence intervals (CIs) were calculated to quantify cohort-specific relative risks, with individuals born during 1960–1964 designated as the reference cohort.

### 2.4. Decomposition and Risk Attribution

To quantify the relative contributions of demographic changes to variations in tuberculosis burden over time, a standard demographic decomposition approach was applied to partition changes in incident cases, deaths, and DALYs into components attributable to population growth, population aging, and changes in age-specific rates. This framework enables the separation of structural demographic effects from epidemiological changes across study periods.

Risk factor attribution was conducted within the comparative risk assessment framework of GBD 2023 using population-attributable fractions (PAFs). Major behavioral and metabolic risk factors relevant to tuberculosis—including tobacco use, high alcohol consumption, high fasting plasma glucose, and high body-mass index (BMI)—were examined. Attributable deaths and DALYs were estimated by applying PAFs to corresponding cause-specific outcomes. Uncertainty intervals (95% UIs) were derived from 1000 posterior draws in accordance with standard GBD methodology.

## 3. Results

### 3.1. Temporal, Age-, and Sex-Specific Trends in the Burden of Tuberculosis in China, 1990–2023

According to GBD 2023 estimates, the overall tuberculosis burden in China declined substantially between 1990 and 2023 across incidence, mortality, and DALYs. However, this long-term downward trend slowed in recent years, with a mild rebound in incidence observed after 2021.

#### 3.1.1. Incidence Trends

**Figure 1:**
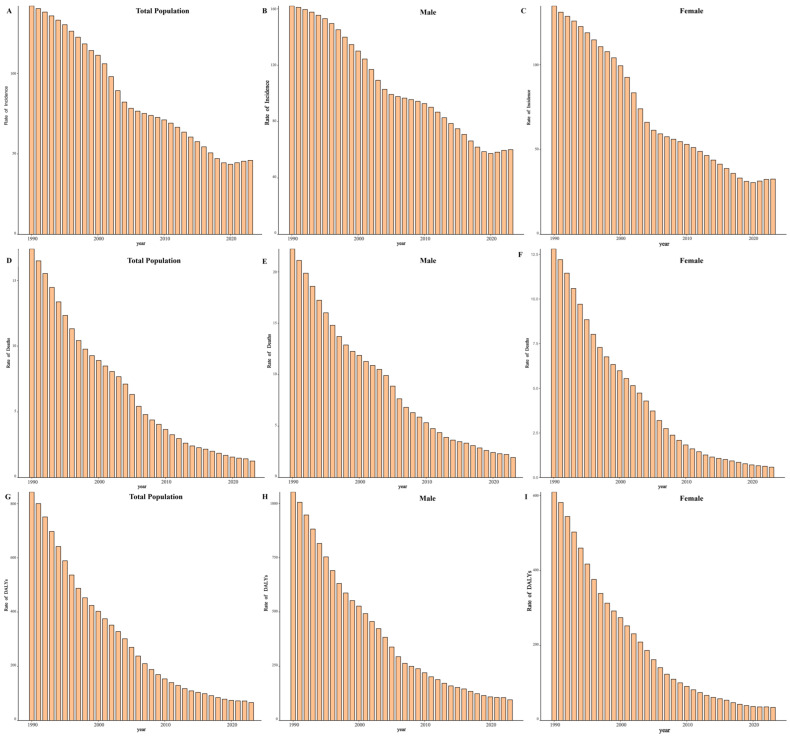
Temporal trends in tuberculosis burden in China, 1990–2023. (**A**–**C**) Age-standardized incidence rates (ASIRs) for total population, males, and females. (**D**–**F**) Age-standardized mortality rates (ASMRs) for total population, males, and females. (**G**–**I**) Age-standardized disability-adjusted life years (DALY) rates (ASDRs) for total population, males, and females. Rates are expressed per 100,000 population based on Global Burden of Disease 2023 estimates.

Between 1990 and 2023, the number of incident TB cases in China decreased from 1,596,222 (95% UI: 1,352,635–1,876,978) to 652,167 (95% UI: 581,880–722,670), representing a 59.14% reduction. Correspondingly, the ASIR declined from 143.66 per 100,000 (95% UI: 123.99–165.71) in 1990 to 38.43 per 100,000 (95% UI: 34.01–42.94) in 2023, with an overall EAPC of −4.49% (95% CI: −4.67 to −4.30) (Figure 1A).

Using 2015 as the baseline year of the WHO End TB Strategy, the ASIR declined from 50.19 per 100,000 in 2015 to 38.43 per 100,000 in 2023, representing a 23.43% reduction and an average annual decrease of 3.28% during this period.

When stratified by time period, the ASIR declined steadily between 1990 and 2021 at an annual rate of −4.59% (95% CI: −4.77 to −4.41). During 2021–2023, ASIR increased at a rate of 1.10% per year (95% CI: 0.42–1.79) (Supplementary Table S1).

Incidence remained consistently higher among males throughout the study period. In 2023, males accounted for 65.02% of new cases, with an ASIR of 48.00 per 100,000 (95% UI: 43.00–53.06), compared with 29.02 per 100,000 (95% UI: 25.32–32.93) among females. The male-to-female ratio of ASIR was approximately 1.65:1 (Figure 1B,C).

**Figure 2:**
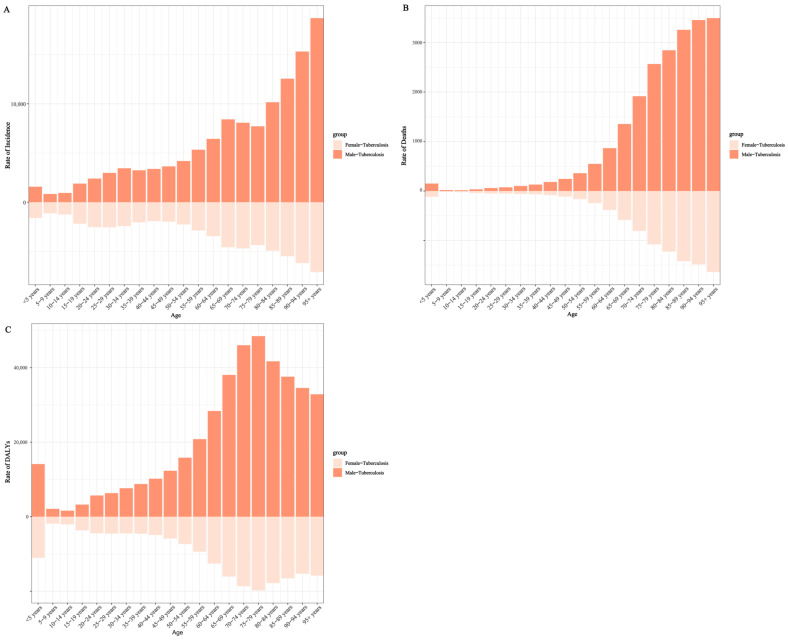
Age- and sex-specific distribution of tuberculosis burden in China, 2023. (**A**) Incidence; (**B**) Mortality; (**C**) Disability-adjusted life years (DALYs). Rates are stratified by age and sex based on GBD 2023 estimates.

The age- and sex-specific distribution of incident tuberculosis in 2023 is illustrated in Figure 2A. Incidence was concentrated among males across nearly all age groups, with relatively higher rates observed in both young adults and older populations.

Age-specific patterns demonstrated a demographic shift toward older populations. The proportion of incident cases among individuals aged ≥60 years increased from 19.17% in 1990 to 37.01% in 2023. In 2023, the 65–69-year age group accounted for the largest share of cases (66,465; 95% UI: 46,922–88,287), representing 10.19% of total incident cases (Supplementary Table S1).

#### 3.1.2. Mortality Trends

The mortality burden of tuberculosis in China declined markedly between 1990 and 2023. The total number of TB-related deaths decreased from 174,628 (95% UI: 128,014–236,420) in 1990 to 24,752 (95% UI: 18,360–34,622) in 2023, representing an 85.83% reduction. The ASMR fell from 19.50 per 100,000 (95% UI: 14.09–26.33) to 1.17 per 100,000 (95% UI: 0.86–1.64), with an overall EAPC of −8.61% (95% CI: −8.85 to −8.37) (Figure 1D).

During the same period (2015–2023), the ASMR declined from 2.14 to 1.17 per 100,000, corresponding to a 45.45% reduction and an average annual decrease of 7.30%.

Between 1990 and 2021, the ASMR declined at an annual rate of −8.67% (95% CI: −8.94 to −8.41). During 2021–2023, mortality declined at a rate of −7.98% (95% CI: −13.18 to −2.48) (Supplementary Table S2).

Mortality remained consistently higher among males. In 2023, males accounted for 73.59% of TB deaths (18,216; 95% UI: 13,344–25,965), compared with 6536 deaths (95% UI: 4118–13,220) among females. The ASMR was 1.79 per 100,000 (95% UI: 1.31–2.56) in males and 0.60 per 100,000 (95% UI: 0.39–1.24) in females, corresponding to a male-to-female ratio of approximately 2.98:1 (Figure 1E,F).

The age- and sex-specific distribution of tuberculosis mortality in 2023 is presented in Figure 2B. Mortality was concentrated among older adults and males across nearly all age groups.

Age-specific patterns demonstrated a demographic shift toward older populations. The proportion of TB deaths occurring among individuals aged ≥60 years increased from 50.27% in 1990 to 70.67% in 2023. In 2023, the 70–74-year age group recorded the highest number of deaths (3548; 95% UI: 2548–5110), accounting for 14.33% of total TB deaths (Supplementary Table S2).

#### 3.1.3. Disability-Adjusted Life Years (DALYs) Trends

The tuberculosis burden expressed in DALYs declined substantially in China between 1990 and 2023. The total number of TB-related DALYs decreased from 7,412,819 person-years (95% UI: 5,716,607–9,784,712) in 1990 to 990,704 person-years (95% UI: 779,739–1,281,147) in 2023, representing an 86.64% reduction. The ASDR declined from 713.39 per 100,000 (95% UI: 545.16–934.90) to 54.23 per 100,000 (95% UI: 42.91–70.14), with an overall EAPC of −8.08% (95% CI: −8.27 to −7.88) (Figure 1G).

Over the same period, the ASDR decreased from 91.04 to 54.20 per 100,000, representing a 40.47% reduction and an average annual decline of 6.28%.

Between 1990 and 2021, the ASDR decreased at an annual rate of −8.19% (95% CI: −8.39 to −7.99). During 2021–2023, the ASDR declined at a rate of −4.57% per year (95% CI: −8.24 to −0.75) (Supplementary Table S3).

DALY burden remained consistently higher among males throughout the study period. In 2023, males accounted for 720,400 DALYs (95% UI: 565,754–937,267), compared with 270,305 DALYs (95% UI: 197,522–444,713) among females. The ASDR was 77.04 per 100,000 (95% UI: 60.98–98.43) in males and 31.73 per 100,000 (95% UI: 23.21–52.11) in females (Figure 1H,I).

The age- and sex-specific distribution of DALYs in 2023 is illustrated in Figure 2C. DALY burden was concentrated among males and older adults across most age groups.

Age-specific patterns demonstrated a demographic shift toward older populations. In 2023, individuals aged ≥60 years accounted for over 45% of total DALYs. The 70–74-year age group recorded the highest DALY burden (110,925 person-years; 95% UI: 84,687–143,982), followed by the 65–69-year groups (Supplementary Table S3).

### 3.2. Age–Period–Cohort Analysis of Tuberculosis Burden in China, 1990–2023

**Figure 3:**
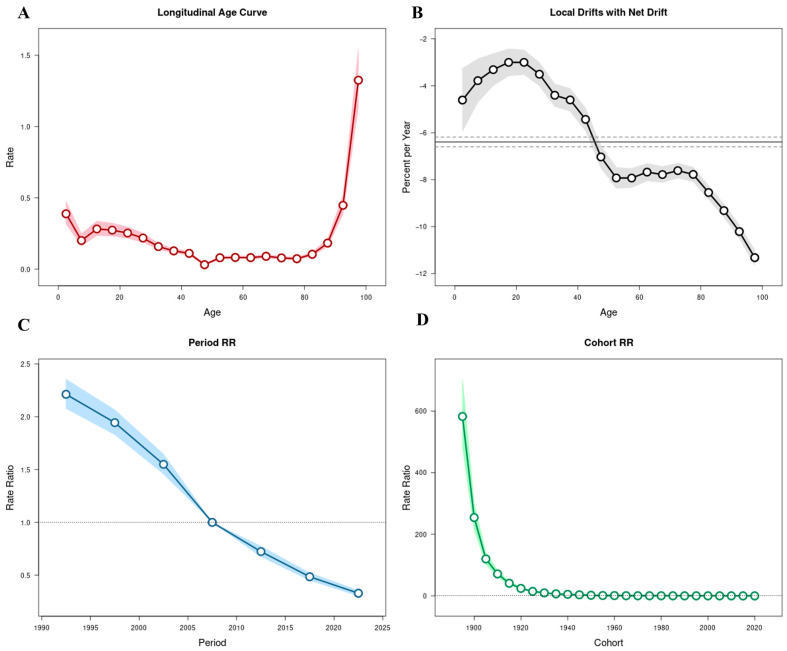
Age–period–cohort (APC) analysis of tuberculosis incidence in China, 1990–2023. (**A**) Longitudinal age curve showing fitted age-specific rates adjusted for period and cohort effects. (**B**) Local drift values (percent change per year) across age groups; the solid line represents local drift estimates for each age group, and the dashed horizontal line indicates the overall net drift. (**C**) Period relative risks (RRs); the solid line represents RR estimates for each period, and the dashed horizontal line at RR = 1 indicates the reference level. (**D**) Cohort relative risks (RRs); the solid line represents RR estimates for each birth cohort, and the dashed horizontal line at RR = 1 indicates the reference cohort. Shaded areas represent 95% confidence intervals (CIs).

**Figure 4:**
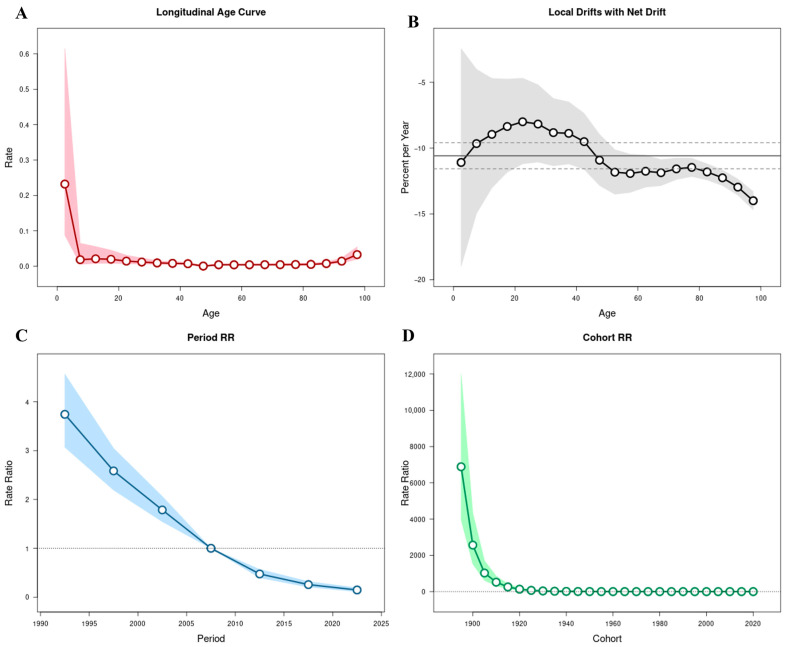
Age–period–cohort (APC) analysis of tuberculosis mortality in China, 1990–2023. (**A**) Longitudinal age curve adjusted for period and cohort effects. (**B**) Local drift values (percent change per year) across age groups; the solid line represents age-specific local drift estimates, and the dashed horizontal line indicates the overall net drift. (**C**) Period relative risks (RRs); the solid line represents RR estimates for each period, and the dashed horizontal line at RR = 1 indicates the reference level. (**D**) Cohort relative risks (RRs); the solid line represents RR estimates for each birth cohort, and the dashed horizontal line at RR = 1 indicates the reference cohort. Shaded areas denote 95% confidence intervals (CIs).

**Figure 5:**
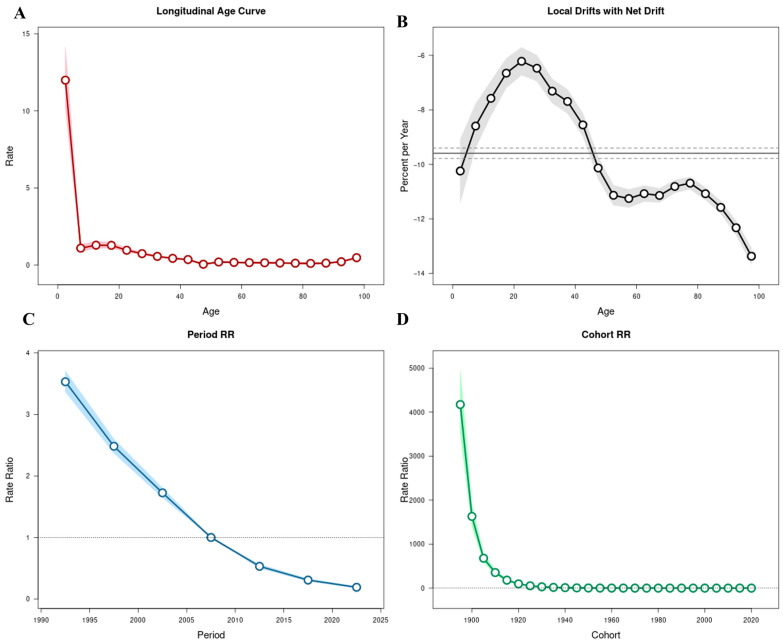
Age–period–cohort (APC) analysis of tuberculosis disability-adjusted life years (DALYs) in China, 1990–2023. (**A**) Longitudinal age curve of age-specific DALY rates adjusted for period and cohort effects. (**B**) Local drift values (percent change per year) across age groups; the solid line represents age-specific local drift estimates, and the dashed horizontal line indicates the overall net drift. (**C**) Period relative risks (RRs); the solid line represents RR estimates for each period, and the dashed horizontal line at RR = 1 indicates the reference level. (**D**) Cohort relative risks (RRs); the solid line represents RR estimates for each birth cohort, and the dashed horizontal line at RR = 1 indicates the reference cohort. Shaded areas represent 95% confidence intervals (CIs).

Using the APC model, the independent effects of age, calendar period, and birth cohort on temporal variations in TB incidence, mortality, and DALYs were estimated (Figure 3, Figure 4 and Figure 5; Supplementary Table S4).

The overall net drift values were −6.39% (95% CI: −6.60 to −6.19) for incidence, −10.59% (95% CI: −11.57 to −9.59) for mortality, and −9.59% (95% CI: −9.78 to −9.40) for DALYs, indicating sustained annual declines across all three indicators between 1990 and 2023. Local drift values were negative across all age groups, although the magnitude of decline varied substantially by age.

Age effects demonstrated distinct patterns across the three metrics. For incidence, RR declined from childhood through middle age, reaching its lowest level among individuals aged 45–49 years (RR = 0.03; 95% CI: 0.03–0.04), followed by a gradual increase in older age groups and peaking among those aged ≥95 years (RR = 1.33; 95% CI: 1.14–1.55) (Figure 3A). Mortality exhibited elevated risks at both extremes of age, with relatively higher risks observed among children under 5 years (RR = 0.23; 95% CI: 0.09–0.62) and a sharp increase among individuals aged ≥90 years (RR = 0.01; 95% CI: 0.01–0.02) (Figure 4A). DALYs showed a comparable age distribution, with higher relative risks in early childhood and advanced age (Figure 5A).

Local drift analysis further revealed heterogeneous rates of decline across age groups. For incidence, annual reductions ranged from −3.00% (95% CI: −3.53 to −2.48) among individuals aged 20–24 years to −11.32% (95% CI: −11.65 to −10.99) among those aged ≥95 years (Figure 3B). Mortality showed steeper annual reductions in older age groups, reaching −14.00% (95% CI: −14.69 to −13.30) among individuals aged ≥95 years, whereas smaller declines were observed in younger adults (e.g., −8.00%; 95% CI: −11.20 to −4.69 in those aged 20−24 years) (Figure 4B). DALYs demonstrated similar patterns, with declines from −10.25% (95% CI: −11.39 to −9.09) among children under 5 years to −13.37% (95% CI: −13.67 to −13.07) in the ≥95-year group (Figure 5B).

Period effects demonstrated a marked and progressive decline in relative risks across successive calendar intervals. Using 2005–2009 as the reference period (RR = 1), the RR for TB incidence decreased from 2.21 (95% CI: 2.08–2.36) in 1990–1994 to 0.33 (95% CI: 0.30–0.36) in 2020–2023 (Figure 3C). Similar reductions were observed for mortality, with RR declining from 3.74 (95% CI: 3.07–4.56) in 1990–1994 to 0.15 (95% CI: 0.11–0.19) in 2020–2023 (Figure 4C), and for DALYs, which decreased from 3.53 (95% CI: 3.37–3.70) to 0.19 (95% CI: 0.18–0.21) over the same period (Figure 5C). These findings indicate consistent downward shifts in period-related risks across all three burden indicators.

Cohort effects revealed progressively lower risks among individuals born in more recent decades. Using the 1960–1964 birth cohort as the reference group (RR = 1), the RR for TB incidence declined from 582.39 (95% CI: 482.37–703.15) in the 1895–1899 cohort to 0.06 (95% CI: 0.03–0.10) in the 2020–2023 cohort (Figure 3D). Mortality and DALYs exhibited comparable cohort gradients, with substantially higher risks in earlier birth cohorts and markedly lower risks among those born after 1970 (Figure 4D and Figure 5D; Supplementary Table S4).

Taken together, the APC analysis demonstrates that the long-term decline in tuberculosis burden in China between 1990 and 2023 was observed across age groups, calendar periods, and successive birth cohorts, with negative net and local drift values confirming sustained annual reductions across all three indicators.

### 3.3. Decomposition of Absolute Changes in Tuberculosis Burden in China, 1990–2023

**Figure 6:**
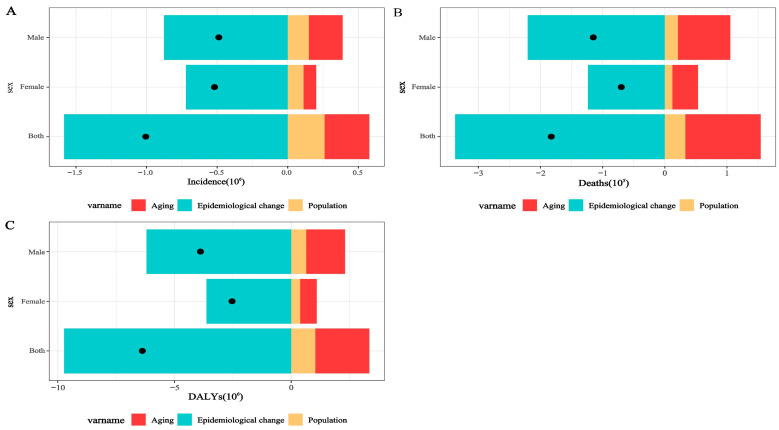
Relative contributions of epidemiologic change, population aging, and population growth to tuberculosis burden in China, 1990–2023. (**A**) Incidence; (**B**) Mortality; (**C**) Disability-adjusted life years (DALYs). Colored bars represent the proportion of each factor’s contribution, and black dots denote the net cumulative effect. Results are stratified by sex based on GBD 2023 estimates.

A three-component decomposition analysis was conducted to quantify the relative contributions of population growth, population aging, and changes in age-specific rates to variations in incident cases, deaths, and DALYs in China between 1990 and 2023. The decomposition was performed using age-specific counts and population data, and results are presented for the total population and stratified by sex (Figure 6).

For tuberculosis incidence, the net reduction over the study period was 1,070,454 cases. Changes in age-specific incidence rates were associated with a decrease of 2,416,863 cases. In contrast, population growth and population aging contributed increases of 712,140 and 634,268 cases, respectively. Relative to the overall net reduction, epidemiologic change accounted for 225.8% of the decline, whereas population growth and population aging offset the reduction by −66.5% and −59.3%, respectively.

For mortality, the net decrease was 208,467 deaths. Epidemiologic change was associated with a reduction of 540,291 deaths, while population aging and population growth contributed increases of 243,510 and 88,314 deaths, respectively. Expressed as proportions of the net change, epidemiologic change accounted for 259.2% of the total decline, whereas population aging and population growth offset the decline by −116.8% and −42.4%, respectively.

Similarly, DALYs declined by 8,236,525 person-years during the study period. Changes in age-specific DALY rates were associated with a reduction of 16,842,026 DALYs, while population aging and population growth contributed increases of 5,096,035 and 3,509,466 DALYs, respectively. As proportions of the net reduction, epidemiologic change accounted for 204.5% of the total decline, whereas population aging and population growth offset the decline by −61.9% and −42.6%, respectively.

Across all three indicators, reductions associated with changes in age-specific rates exceeded the net observed decline, whereas demographic shifts—particularly population aging—partially counterbalanced these reductions. These findings indicate that although improvements in tuberculosis control substantially reduced age-specific risks, demographic transition has increasingly influenced the overall burden structure..

### 3.4. Risk Factor Attribution of Tuberculosis Burden in China, 1990–2023

**Figure 7:**
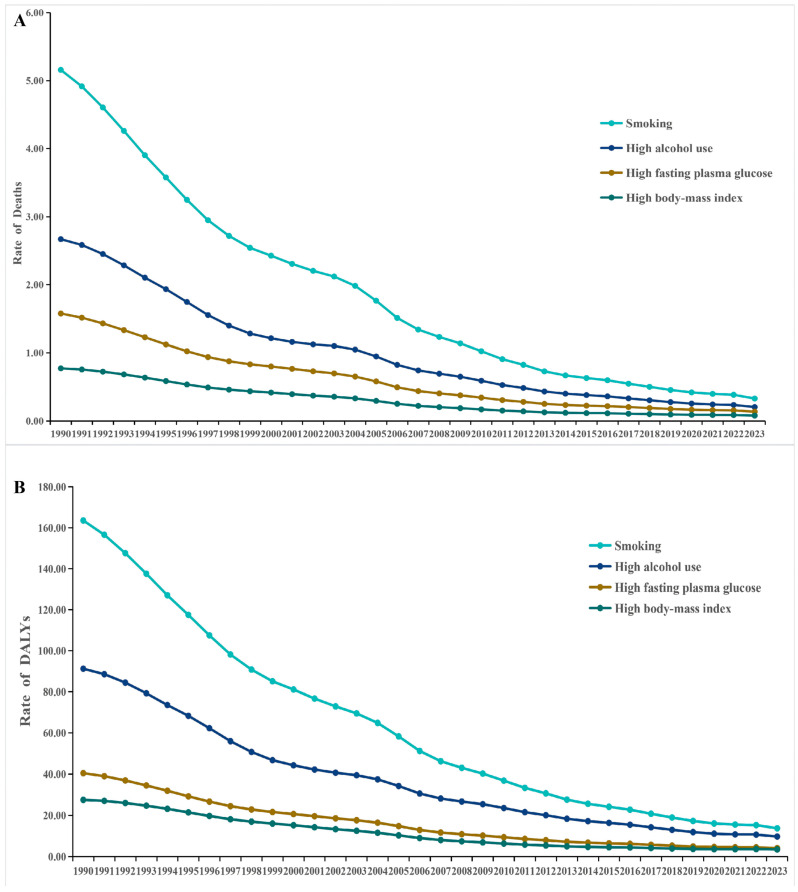
Temporal trends in tuberculosis burden attributable to selected risk factors in China, 1990–2023. (**A**) Age-standardized mortality rates attributable to smoking, high alcohol use, high fasting plasma glucose, and high body-mass index (BMI). (**B**) Age-standardized disability-adjusted life year (DALY) rates attributable to the same risk factors. Rates are expressed per 100,000 population and estimated based on the Global Burden of Disease (GBD) 2023 comparative risk assessment framework.

The comparative risk assessment analysis identified smoking, high alcohol use, high fasting plasma glucose, and high BMI as the four principal modifiable risk factors contributing to TB mortality and DALYs in China between 1990 and 2023 (Figure 7).

Across the study period, age-standardized mortality and DALY rates attributable to all four risk factors demonstrated consistent downward trends. Smoking remained the leading attributable risk factor throughout the entire period. The age-standardized mortality rate attributable to smoking declined from approximately 5.16 per 100,000 in 1990 to 0.33 per 100,000 in 2023. Similarly, smoking-attributable DALY rates decreased from 163.56 per 100,000 to 13.75 per 100,000 over the same period (Figure 7A,B).

High alcohol use represented the second largest contributor to TB burden. Mortality attributable to alcohol use declined from approximately 2.67 per 100,000 in 1990 to 0.20 per 100,000 in 2023, while DALY rates decreased from 91.22 to 9.76 per 100,000.

High fasting plasma glucose and high BMI contributed comparatively smaller but measurable proportions of TB burden. In 2023, mortality attributable to high fasting plasma glucose was 0.21 per 100,000 among males and 0.07 per 100,000 among females, while corresponding DALY rates were 6.41 and 1.99 per 100,000, respectively. High BMI contributed 0.12 per 100,000 in male mortality and 0.04 per 100,000 in female mortality in 2023, with DALY rates of 5.07 and 1.93 per 100,000, respectively (Figure 7A,B).

**Figure 8:**
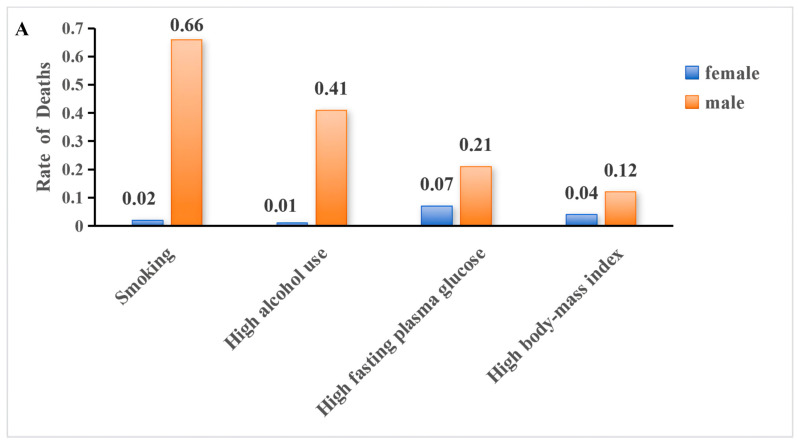

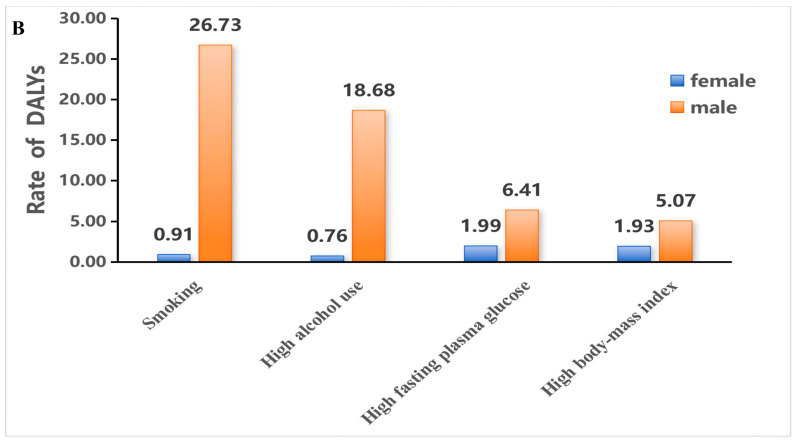
Sex-specific tuberculosis burden attributable to selected risk factors in China, 2023. (**A**) Age-standardized mortality rates attributable to smoking, high alcohol use, high fasting plasma glucose, and high body-mass index (BMI), stratified by sex. (**B**) Age-standardized disability-adjusted life year (DALY) rates attributable to the same risk factors, stratified by sex. Rates are expressed per 100,000 population based on GBD 2023 estimates.

Sex-stratified analyses revealed marked disparities. In 2023, the attributable burden of smoking and high alcohol use was substantially higher among males than females. Smoking accounted for 0.66 per 100,000 in male mortality compared with 0.02 per 100,000 in females, while alcohol use accounted for 0.41 per 100,000 in males versus 0.01 per 100,000 in females. For DALYs, smoking-attributable rates were 26.73 per 100,000 in males compared with 0.91 per 100,000 in females, and alcohol-attributable DALYs were 18.68 per 100,000 in males versus 0.76 per 100,000 in females (Figure 8).

Although high fasting plasma glucose and high BMI contributed lower absolute rates than behavioral risk factors, their relative contribution among females was proportionally greater than that observed for smoking and alcohol use. Overall, smoking remained the dominant attributable risk factor for both mortality and DALYs, followed by high alcohol use, while metabolic risk factors contributed smaller but non-negligible components of TB burden in 2023.

## 4. Discussion

### 4.1. Interpretation of Long-Term Trends

This study provides an updated reassessment of TB burden in China from 1990 to 2023 based on the latest GBD 2023 estimates. Over the past three decades, age-standardized incidence, mortality, and DALY rates declined substantially, reflecting sustained and system-wide progress in tuberculosis prevention and control. These reductions are consistent with global downward trends documented by the WHO, which have shown a marked decline in TB mortality since the early 2000s, including substantial reductions in the Western Pacific Region [2,23]. Similar long-term declines have also been observed in successive GBD analyses evaluating progress toward the WHO End TB Strategy milestones [23], and WHO assessments attribute these reductions to expanded case detection, standardized treatment strategies, and strengthening of national TB programs [2]. Despite substantial long-term progress since 1990, the post-2015 rate of decline—particularly in incidence—remains slower than the trajectory required to achieve the 2035 End TB Strategy milestones.

The persistently negative net drift and local drift values identified in our age–period–cohort analysis further indicate that the decline in TB burden in China was not confined to specific subgroups but occurred broadly across age strata. This pattern suggests sustained improvements in both transmission control and case-fatality reduction, likely reflecting the cumulative effects of nationwide Directly Observed Treatment, Short-Course (DOTS) expansion and progressive strengthening of China’s public health system over the past two decades [24,25].

However, the temporal trajectory was not strictly linear. The modest increase in age-standardized incidence observed after 2021, together with a slower decline in mortality and DALYs, suggests a recent deceleration in progress. Similar disruptions were documented globally during the COVID-19 pandemic. WHO reports described sharp declines in TB case notifications in 2020, followed by partial recovery in subsequent years as essential services resumed [2]. Early modeling studies projected that COVID-19–related service interruptions could lead to substantial excess TB cases and deaths due to delayed diagnosis and treatment [26]. Empirical analyses based on national surveillance data in China likewise identified significant temporary declines in reported TB notifications during the pandemic period, with gradual normalization as health services stabilized [27]. Taken together, these findings suggest that the post-2021 increase in incidence in China most plausibly reflects delayed case detection and service recovery rather than a fundamental resurgence of transmission.

Importantly, our decomposition analysis demonstrated that epidemiologic improvements accounted for the majority of the long-term reduction in TB burden, while demographic change—particularly population aging—exerted an opposing but smaller effect. This dual dynamic indicates that although transmission intensity and case-fatality have declined substantially, structural population shifts are increasingly influencing national burden patterns. In settings where TB incidence has declined to intermediate or relatively low levels, demographic aging has been recognized as an important modifier of overall disease burden, as reactivation of LTBI among older adults increasingly contributes to incident cases [28,29]. The slowing pace of decline observed in recent years may therefore reflect a transition toward a more complex epidemiologic phase shaped by demographic and structural determinants, underscoring the need to prioritize high-risk groups—particularly older adults with comorbidities and close contacts of infectious TB cases—for LTBI screening and preventive interventions [30,31].

Overall, China’s TB epidemic appears to have entered a stage characterized not by widespread transmission escalation, but by moderated long-term gains within a changing demographic and health-system context. Sustaining further reductions will require maintaining transmission control while strengthening system resilience and adapting strategies to evolving structural pressures.

### 4.2. Demographic and Risk Transition of Tuberculosis Burden

Beyond the overall temporal decline, the present analysis highlights a pronounced demographic restructuring of tuberculosis burden in China. The increasing proportion of cases, deaths, and DALYs occurring among individuals aged ≥60 years reflects a shift in disease concentration toward older populations. This pattern parallels observations in other rapidly aging East Asian countries, where tuberculosis burden has progressively migrated to older age groups despite sustained reductions in overall incidence [32,33]. Population aging has been recognized as a key modifier of TB epidemiology in low- and intermediate-incidence settings, as cumulative lifetime exposure and age-related immune vulnerability elevate the relative contribution of older adults to national burden profiles [34,35].

Sex disparities also remained a persistent feature of TB epidemiology in China. Males exhibited consistently higher incidence, mortality, and DALY rates throughout the study period. Similar male predominance has been widely documented at both global and regional levels, with men accounting for approximately two-thirds of TB cases worldwide [2,36]. Multiple studies have attributed this disparity to differences in health-seeking behavior, occupational exposure, and higher prevalence of behavioral risk factors among men [37,38,39]. The stronger impact of smoking- and alcohol-attributable burden observed in males in our analysis aligns with prior global comparative risk assessments, which identify tobacco use and alcohol consumption as major contributors to sex differentials in TB outcomes [36,40,41].

In addition to behavioral risks, nutritional and metabolic factors remain important components of TB epidemiology. Undernutrition and low BMI are well-established risk factors for active tuberculosis globally, reflecting the critical role of nutritional status in host immune competence [42,43,44,45]. Although high fasting plasma glucose and high BMI contributed smaller proportions of attributable burden compared with smoking and alcohol use in our analysis, the coexistence of tuberculosis with diabetes and other metabolic disorders has gained growing attention in aging populations [46,47,48]. The bidirectional interaction between TB and diabetes has been recognized as an important challenge in countries undergoing rapid epidemiologic transition, including China [49,50]. Our findings suggest that metabolic vulnerability may represent an additional layer influencing future TB dynamics within an aging demographic framework.

Taken together, these demographic and risk-related patterns indicate that the structure of TB burden in China is evolving. While transmission control has driven long-term reductions, age composition, sex-specific risk exposures, and the rising prevalence of metabolic conditions are increasingly shaping the epidemiologic landscape. Understanding this structural transition is essential for interpreting recent trends and anticipating future burden trajectories.

### 4.3. Strengths and Limitations

This study has several notable strengths. By utilizing the most recent Global Burden of Disease 2023 estimates, it provides an updated and post-pandemic reassessment of tuberculosis burden in China over a 33-year period. The integration of age–period–cohort modeling with demographic decomposition enabled a multidimensional evaluation of temporal dynamics, allowing us to distinguish age, period, and cohort effects while quantitatively partitioning epidemiologic and demographic contributions to burden change. In addition, the incorporation of comparative risk assessment offered further insight into the behavioral and metabolic determinants underlying long-term trends. Together, these complementary analytic approaches enhance the robustness and interpretability of the findings.

Several limitations should be acknowledged. First, GBD estimates are derived from heterogeneous surveillance and vital registration sources and rely on statistical modeling to address data gaps and inconsistencies. Although Bayesian meta-regression frameworks improve internal consistency across age, sex, location, and time, residual uncertainty may persist, particularly for age-specific and risk-attributable estimates. Second, this analysis was conducted at the national level and does not capture potential subnational heterogeneity in tuberculosis burden across provinces or urban–rural settings. Third, the comparative risk assessment framework assumes stable exposure–outcome relationships and may not fully account for complex interactions among demographic, behavioral, and metabolic determinants. Finally, the study did not distinguish between drug-susceptible and drug-resistant tuberculosis or between HIV-associated and non-HIV-associated cases, which may exhibit distinct epidemiologic patterns.

Despite these limitations, the present analysis provides a comprehensive overview of long-term tuberculosis burden dynamics in China and offers a structured interpretation of demographic and risk-related transitions within a nationally representative framework.

## 5. Conclusions

This study provides a comprehensive reassessment of the TB burden in China from 1990 to 2023 based on the latest GBD 2023 estimates. Over the past three decades, age-standardized incidence, mortality, and DALY rates have declined substantially, reflecting sustained progress in transmission control, case detection, and treatment effectiveness. However, the recent deceleration in incidence reduction, particularly after 2021, suggests that China’s TB epidemic has entered a more complex epidemiologic phase.

Beyond overall declines, the burden structure has shifted markedly. Population aging, persistent sex disparities, and evolving behavioral and metabolic risk profiles are increasingly shaping national TB dynamics. Although epidemiologic improvements remain the primary driver of long-term reductions, demographic transitions—especially rapid aging—are progressively modifying disease patterns and may constrain future gains.

Taken together, these findings indicate that sustaining progress toward TB elimination in China will require not only continued transmission control but also demographic- and risk-sensitive strategies. Strengthening health system resilience, integrating TB management with chronic disease care, and prioritizing high-risk populations will be essential to maintain momentum in the context of ongoing population aging and structural transition.

## Data Availability

This study follows the Guidelines for Accurate and Transparent Health Estimates Reporting (GATHER). All data used in this analysis were obtained from the GBD 2023, coordinated by the IHME, University of Washington. The datasets analyzed are publicly available through the Global Health Data Exchange (GHDx) portal: https://ghdx.healthdata.org/gbd-2023 (accessed on 1 January 2026).

## Supplementary Materials

The following supporting information can be downloaded at: https://www.mdpi.com/article/10.3390/pathogens15030295/s1, Table S1: Temporal and demographic patterns of tuberculosis incidence in China, 1990–2023; Table S2: Temporal and demographic patterns of tuberculosis mortality in China, 1990–2023; Table S3: Temporal and demographic patterns of tuberculosis disability-adjusted life years (DALYs) in China, 1990–2023; Table S4: Estimated parameters of the age–period–cohort (APC) model for tuberculosis incidence, mortality, and DALYs in China, 1990–2023.

## Abbreviations

The following abbreviations are used in this manuscript:

TB	Tuberculosis
GBD	Global Burden of Disease
IHME	Institute for Health Metrics and Evaluation
WHO	World Health Organization
DALY	Disability-Adjusted Life Year
ASR	Age-Standardized Rate
ASIR	Age-Standardized Incidence Rate
ASMR	Age-Standardized Mortality Rate
ASDR	Age-Standardized DALY Rate
EAPC	Estimated Annual Percentage Change
RR	Rate Ratio
CI	Confidence Interval
UI	Uncertainty Interval
APC	Age–Period–Cohort
IE	Intrinsic Estimator
DOTS	Directly Observed Treatment, Short-Course
MDR-TB	Multidrug-Resistant Tuberculosis
