# Supplementary material for: Long-Term Trends and Determinants of Tuberculosis Burden in China, 1990–2023: Insights from the Global Burden of Disease Study 2023
Source: Pathogens. 2026 Mar 8;15(3):295. doi: 10.3390/pathogens15030295 (PMC13029187; doi:10.3390/pathogens15030295)
Supplement: Supplementary file 1 [file pathogens-15-00295-s001.zip › pathogens-4122566-supplementary.pdf]

Supplementary Table S1. Temporal and demographic patterns of tuberculosis incidence in China, 1990–2023.

Group	Number of incidences			ASIR per 100000			EAPC (95%CI)		
	1990(95%UI)	2021(95%UI)	2023(95%UI)	1990(95%UI)	2021(95%UI)	2023(95%UI)	1990—2021	2021—2023	1990–2023
Total	1596222 (1352635—1876978)	628097 (558414—695358)	652167 (581880—722670)	143.66 (123.99—165.71)	37.59 (33.17—41.73)	38.43 (34.01—42.94)	-4.59 (-4.77—-4.41)	1.10 (0.42—1.79)	-4.49 (-4.67—-4.30)
Sex									
Male	871625 (742672—1014064)	408127 (363921—450911)	424052 (380710—468251)	159.85 (139.85—183.13)	46.98 (41.99—52.04)	48.00 (43.00—53.06)	-4.16 (-4.34—-3.98)	1.08 (0.76—1.39)	-4.09 (-4.26—-3.92)
Female	724597 (604250—866296)	219970 (195089—246786)	228115 (203278—255519)	129.27 (109.64—150.97)	28.41 (24.89—32.16)	29.02 (25.32—32.93)	-5.24 (-5.43—-5.04)	1.07 (-0.24—2.39)	-5.08 (-5.30—-4.87)
Age									
<5 years	101989 (78500—132938)	11429 (9037—14922)	8998(7182—11590)	92.01 (70.82—119.93)	14.70(11.62—19.19)	14.48 (11.55—18.64)	-6.06 (-6.35—-5.76)	-0.77 (-1.48—-0.06)	-6.05 (-6.31—-5.79)
5—9 years	69358 (45071—98876)	11622 (7305—17483)	12202 (7513—18443)	67.63 (43.95—96.41)	13.47 (8.47—20.27)	13.75(8.46—20.78)	-5.01 (-5.34—-4.69)	1.02 (0.72—1.31)	-4.91 (-5.21—-4.61)
10—14 years	146370 (85890—222420)	22389 (14492—32222)	24392 (15650—35551)	115.25(67.63—175.13)	29.91 (19.36—43.05)	30.28 (19.43—44.13)	-4.49 (-4.81—-4.18)	0.61 (-0.09—1.31)	-4.34 (-4.64—-4.03)
15—19 years	178152(106939—261894)	28516 (18043—40000)	28409.542 (17767.467—39999.359)	134.59(80.79—197.85)	39.06 (24.71—54.79)	40.15 (25.11—56.53)	-4.23 (-4.49—-3.96)	1.39 (0.12—2.67)	-4.04 (-4.32—-3.76)
20—24 years	148999 (93202—220592)	37336 (25373—51796)	34912(23340—48828)	135.24 (84.60—200.22)	43.15 (29.32—59.86)	44.76 (29.93—62.61)	-3.97 (-4.16—-3.78)	1.85 (0.77—2.95)	-3.85 (-4.04—-3.66)
25—29 years	122186 (78211—177050)	54738 (36226—77267)	50678 (33832—71984)	138 (88—200)	45.14 (29.87—63.72)	47.18 (31.50—67.01)	-3.87 (-4.07—-3.67)	2.24 (1.05—3.43)	-3.78 (-3.98—-3.59)
30—34 years	118398 (71747—169325)	41443 (27683—55372)	47517 (30851—63801)	129 (78—185)	39.18 (26.17—52.34)	40.19 (26.10—53.97)	-4.14 (-4.33—-3.94)	1.29 (-0.14—2.74)	-4.05 (-4.23—-3.86)
35—39 years	86989 (52216—124752)	36250(25065—49278)	36367 (24891—49419)	129(78—185)	39.56 (27.35—53.77)	39.99 (27.37—54.34)	-4.19 (-4.37—-4.01)	0.54 (-0.77—1.88)	-4.09 (-4.27—-3.92)

40—44 years	72556(44488—104723)	46282 (31498—62059)	42709 (28798—56942)	140 (86—202)	41.96 (28.55—56.26)	42.82 (28.87—57.09)	-4.23 (-4.39—-4.06)	1.02 (-0.02—2.07)	-4.12 (-4.29—-3.95)
45—49 years	72998 (49698—101690)	8992 (6077—13162)	9473 (6335—13881)	70 (48—97)	9.41 (6.36—13.77)	9.36 (6.26—13.72)	-6.28 (-6.57—-5.98)	-0.25 (-0.68—0.18)	-6.16 (-6.44—-5.89)
50—54 years	78241 (48326—114240)	54480 (38625—73569)	54964(38636—74025)	164(101—239)	45.06 (31.95—60.85)	46.42 (32.63—62.52)	-4.60 (-4.80—-4.40)	1.50 (1.26—1.75)	-4.47 (-4.67—-4.27)
55—59 years	93975 (58246—138180)	55137 (38878—73748)	60177 (41263—80604)	216 (134—318)	50.20 (35.39—67.14)	50.90 (34.90—68.18)	-5.33 (-5.60—-5.06)	0.70(0.62—0.78)	-5.20 (-5.46—-4.94)
60—64 years	93151 (58647—131758)	42968 (30820—56964)	48837 (34881—65163)	263 (165—372)	58.83 (42.20—77.99)	59.98 (42.84—80.03)	-5.36 (-5.60—-5.12)	0.97 (0.72—1.21)	-5.24 (-5.47—-5.01)
65—69 years	87246 (54733—121076)	64736 (45589—86258)	66465 (46922—88287)	318 (200—442)	83.92 (59.10—111.81)	87.10 (61.49—115.69)	-4.58 (-4.81—-4.35)	1.88 (1.62—2.14)	-4.52 (-4.73—-4.31)
70—74 years	60372 (41551—85161)	43940 (32163—56419)	51398 (37217—66633)	320(220—451)	82.31 (60.25—105.69)	84.18(60.96—109.14)	-4.50 (-4.74—-4.26)	1.13 (0.82—1.44)	-4.46 (-4.68—-4.25)
75—79 years	35169 (22330—49924)	26123 (18606—34999)	29033(20499—38985)	308 (195—437)	78.79 (56.12—105.56)	78.92 (55.72—105.97)	-4.45 (-4.67—-4.24)	0.09 (0.00—0.17)	-4.41 (-4.60—-4.22)
80—84 years	19987 (14822—26417)	21529 (16734—26887)	23090 (17882—28808)	358 (265—473)	101.67 (79.03—126.98)	102.78 (79.60—128.24)	-4.16 (-4.37—-3.95)	0.55 (0.07—1.03)	-4.11 (-4.30—-3.91)
85—89 years	8012 (6089—10277)	13514 (10965—16114)	14804 (12052—17600)	401 (305—515)	116.43(94.47—138.82)	118.70 (96.63—141.12)	-4.06 (-4.30—-3.83)	0.97 (0.48—1.46)	-4.01 (-4.23—-3.80)
90—94 years	1708 (1236—2261)	5186 (4119—6407)	5938(4634—7410)	454 (328—600)	130.00 (103.24—160.61)	131.12 (102.33—163.64)	-4.11(-4.35—-3.87)	0.43 (-0.34—1.21)	-4.06 (-4.28—-3.85)
95+ years	364 (223—515)	1486(1061—2028)	1802 (1254—2479)	555(340—787)	143.72 (102.54—196.05)	145.15(101.03—199.64)	-4.371(-4.58—-4.16)	0.50 (-0.31—1.31)	-4.29 (-4.49—-4.09)

ASIR: age-standardized incidence rate; EAPC: estimated annual percentage change; CI: confidence interval; UI: uncertainty interval.

Supplementary Table S2. Temporal and demographic patterns of tuberculosis mortality in China, 1990–2023.

Group	Number of deaths			ASMR per 100000			EAPC (95%CI)		
	1990(95%UI)	2021(95%UI)	2023(95%UI)	1990(95%UI)	2021(95%UI)	2023(95%UI)	1990—2021	2021—2023	1990–2023
Total	174628 (128014—236420)	27769 (23054—33483)	24752 (18360—34622)	19.50 (14.09—26.33)	1.38 (1.14—1.67)	1.17 (0.86—1.64)	-8.67(-8.94—-8.41)	-7.98(-13.18—-2.48)	-8.61 (-8.85—-8.37)
Sex									
Male	109420 (74362—151542)	20689(16526—25965)	18216 (13344—25965)	25.73 (16.90—35.87)	2.13 (1.69—2.72)	1.79 (1.31—2.56)	-8.15 (-8.42—-7.88)	-8.43(-14.43—-2.01)	-8.11 (-8.35—-7.87)
Female	65208 (37402—95138)	7080 (5100—10782)	6536 (4118—13220)	14.13(8.05—20.67)	0.69(0.51—1.05)	0.60 (0.39—1.24)	-9.87(-10.16—-9.58)	-6.70 (-9.64—-3.66)	-9.76(-10.03—-9.49)
Age									
<5 years	13135 (8657—16361)	183 (155—224)	132 (104—183)	11.85 (7.81—14.76)	0.24 (0.20—0.29)	0.21 (0.17—0.29)	-12.21 (-12.88—-11.54)	-5.12 (-8.97—-1.10)	-12.37 (-12.98—-11.77)
5—9 years	1719 (1179—2452)	69 (48—97)	66 (42—114)	1.68(1.15—2.39)	0.08 (0.06—0.11)	0.07 (0.05—0.13)	-9.61 (-9.76—-9.46)	-3.55 (-6.20—-0.83)	-9.52 (-9.67—-9.36)
10—14 years	4179 (2960—5982)	138 (96—188)	132 (84—222)	3.29 (2.33—4.71)	0.18 (0.13—0.25)	0.16 (0.10—0.28)	-9.50 (-9.76—-9.25)	-5.50 (-11.76—1.19)	-9.30 (-9.58—-9.03)
15—19 years	6248 (4353—8792)	235 (163—319)	195 (122—289)	4.72 (3.29—6.64)	0.32 (0.22—0.44)	0.28 (0.17—0.41)	-9.14 (-9.51—-8.77)	-7.55 (-15.20—0.80)	-8.93 (-9.30—-8.56)
20—24 years	5379 (3850—7644)	335 (236—451)	276 (163—418)	4.88 (3.49—6.94)	0.39 (0.27—0.52)	0.35 (0.21—0.54)	-8.49 (-8.83—-8.15)	-4.38 (-9.80—1.37)	-8.36 (-8.68—-8.04)
25—29 years	5578 (3905—7614)	555 (405—732)	449 (284—694)	6.30 (4.41—8.60)	0.46 (0.33—0.60)	0.42 (0.26—0.65)	-8.63 (-9.01—-8.25)	-4.40 (-9.57—1.06)	-8.55 (-8.89—-8.21)
30—34 years	7178 (4854—10315)	625 (472—838)	592 (386—876)	7.84 (5.30—11.27)	0.59 (0.45—0.79)	0.50 (0.33—0.74)	-8.51 (-8.83—-8.19)	-7.86 (-14.16—-1.11)	-8.45 (-8.74—-8.16)
35—39 years	7488 (5115—10694)	755 (574—987)	636 (414—916)	11.13 (7.60—15.89)	0.82 (0.63—1.08)	0.70 (0.46—1.01)	-8.50 (-8.75—-8.25)	-7.88 (-13.14—-2.30)	-8.41 (-8.65—-8.18)

40—44 years	7933 (5482—11016)	1279 (976—1637)	995 (673—1481)	15.33 (10.59—21.29)	1.16 (0.89—1.48)	1.00 (0.68—1.48)	-8.25 (-8.42—-8.08)	-7.24 (-11.57—-2.71)	-8.17 (-8.33—-8.00)
45—49 years	1727 (1136—2460)	58 (40—82)	56 (34—88)	1.65 (1.09—2.35)	0.06 (0.04—0.09)	0.06 (0.03—0.09)	-10.49 (-10.74—-10.24)	-4.11 (-4.46—-3.75)	-10.43 (-10.65—-10.20)
50—54 years	11021 (7914—15675)	1903 (1450—2378)	1591 (1066—2307)	23.04 (16.54—32.77)	1.57 (1.20—1.97)	1.34 (0.90—1.95)	-8.61 (-8.77—-8.44)	-7.61 (-12.09—-2.91)	-8.51 (-8.68—-8.35)
55—59 years	15259 (10786—21997)	2406 (1908—3009)	2140 (1434—3233)	35.08 (24.80—50.57)	2.19 (1.74—2.74)	1.81 (1.21—2.73)	-9.24 (-9.52—-8.95)	-9.08 (-15.67—-1.97)	-9.09 (-9.37—-8.80)
60—64 years	19108 (13914—26928)	2360 (1905—2951)	2219 (1533—3134)	53.90 (39.25—75.96)	3.23 (2.61—4.04)	2.73 (1.88—3.85)	-9.31 (-9.60—-9.02)	-8.16 (-13.17—-2.85)	-9.19 (-9.46—-8.92)
65—69 years	21984 (15695—30336)	3639 (3017—4395)	3063 (2266—4379)	80.20 (57.26—110.67)	4.72 (3.91—5.70)	4.01 (2.97—5.74)	-9.12(-9.41—-8.83)	-7.75(-12.37—-2.89)	-9.10 (-9.35—-8.84)
70—74 years	20117 (13760—28365)	3745 (3161—4669)	3548 (2548—5110)	106.57 (72.90—150.27)	7.02 (5.92—8.75)	5.81 (4.17—8.37)	-8.86 (-9.20—-8.52)	-8.98 (-14.65—-2.94)	-8.847 (-9.148—-8.546)
75—79 years	15503 (10618—21657)	3575 (2924—4296)	3256 (2257—4725)	135.67 (92.92—189.53)	10.78 (8.82—12.96)	8.85 (6.14—12.84)	-8.18 (-8.45—-7.91)	-9.39 (-14.97—-3.44)	-8.18 (-8.42—-7.94)
80—84 years	7538 (4942—11334)	3007 (2385—3738)	2711 (1811—3952)	134.99 (88.51—202.97)	14.20 (11.26—17.65)	12.07 (8.06—17.59)	-7.40 (-7.66—-7.14)	-7.82 (-12.30—-3.11)	-7.37 (-7.61—-7.14)
85—89 years	2898 (1858—4432)	1974 (1484—2545)	1800 (1112—2672)	145.17 (93.10—222.04)	17.00 (12.78—21.92)	14.43 (8.91—21.43)	-7.12 (-7.39—-6.85)	-7.87 (-15.86—0.87)	-7.06 (-7.30—-6.81)
90—94 years	533 (356—790)	747 (522—1029)	705 (452—1040)	141.56 (94.63—209.76)	18.74 (13.08—25.79)	15.58 (9.99—22.96)	-6.85 (-7.17—-6.52)	-8.82 (-12.03—-5.48)	-6.79 (-7.09—-6.50)
95+ years	104 (66—155)	183 (122—265)	189 (113—287)	159.03 (100.45—237.23)	17.65 (11.78—25.59)	15.26 (9.11—23.13)	-7.28 (-7.61—-6.95)	-7.02 (-9.68—-4.28)	-7.18 (-7.48—-6.88)

ASDR: age-standardized DALY rate; EAPC: estimated annual percentage change; CI: confidence interval; UI: uncertainty interval.

Supplementary Table S3. Temporal and demographic patterns of tuberculosis disability-adjusted life years (DALYs) in China, 1990–2023.

Group	Number of DALYs			ASDR per 100000			EAPC(95%CI)		
	1990(95%UI)	2021(95%UI)	2023(95%UI)	1990(95%UI)	2021(95%UI)	2023(95%UI)	1990—2021	2021—2023	1990–2023
Total	7412819 (5716607—9784712)	1067621 (900579—1253955)	990704 (779739—1281147)	713.39 (545.16—934.90)	59.55 (50.30—70.35)	54.23 (42.91—70.14)	-8.19 (-8.39—-7.99)	-4.57 (-8.24—-0.75)	-8.08 (-8.27—-7.88)
Sex									
Male	4534693 (3371670—6144202)	783842 (650767—968627)	720400 (565754—937267)	883.18 (639.48—1196.74)	85.58 (71.24—105.08)	77.04 (60.98—98.43)	-7.68 (-7.88—-7.48)	-5.12 (-9.58—-0.44)	-7.60(-7.78—-7.41)
Female	2878127(1815452—3885212)	283779(220339—382497)	270305 (197522—444713)	550.00 (345.01—745.41)	33.93 (26.44—45.77)	31.73 (23.21—52.11)	-9.19 (-9.41—-8.97)	-3.29 (-5.01—-1.54)	-9.04 (-9.27—-8.81)
Age									
<5 years	1206421 (805987—1495872)	22539 (18447—27060)	16614 (13102—22172)	1088.40 (727.14—1349.53)	28.99 (23.73—34.80)	26.73 (21.08—35.667)	-11.48 (-12.04—-10.91)	-3.98 (-7.10—-0.76)	-11.56 (-12.06—-11.05)
5—9 years	157701 (115838—218177)	9633 (7004—13005)	9753 (6590—14607)	153.77 (112.95—212.74)	11.17(8.12—15.07)	10.987 (7.423—16.46)	-8.18 (-8.38—-7.98)	-0.80 (-3.99—2.49)	-8.07 (-8.26—-7.87)
10—14 years	346980(250674—472985)	14660 (10783—18783)	14794 (10305—21450)	273.21 (197.38—372.43)	19.59 (14.41—25.10)	18.36 (12.79—26.63)	-8.56 (-8.75—-8.36)	-3.17 (-8.49—2.45)	-8.45 (-8.64—-8.27)
15—19 years	497183 (365702—679375)	33058 (23778—45235)	30357 (21180—43284)	375.60 (276.27—513.24)	45.28 (32.57—61.96)	42.90 (29.93—61.17)	-7.29 (-7.59—-7.00)	-2.66 (-7.26—2.17)	-7.05(-7.37—-6.72)
20—24 years	401698(298624—542979)	44140 (32038—59338)	38858 (26495—55364)	364.60 (271.05—492.84)	51.01 (37.03—68.58)	49.82 (33.97—70.99)	-6.62 (-6.82—-6.42)	-1.17 (-4.15—1.89)	-6.47 (-6.68—-6.25)

25—29 years	369216 (277804—489324)	61060 (45665—80516)	51381 (36403—72985)	417.19 (313.90—552.91)	50.35 (37.66—66.39)	47.83 (33.89—67.95)	-7.02 (-7.26—-6.77)	-2.53 (-6.21—1.29)	-6.92 (-7.15—-6.69)
30—34 years	437646 (318695—600851)	60054 (46498—76907)	61929 (45420—83978)	477.96 (348.06—656.21)	56.77 (43.95—72.70)	52.39 (38.42—71.04)	-7.04 (-7.20—-6.87)	-3.94 (-8.15—0.47)	-6.93 (-7.10—-6.76)
35—39 years	394424 (283697—560399)	52877 (40816—67343)	47182 (34495—64188)	586.17 (421.61—832.83)	57.70 (44.54—73.48)	51.88 (37.93—70.58)	-7.62 (-7.80—-7.44)	-5.18(-9.73—-0.39)	-7.50 (-7.68—-7.31)
40—44 years	376862 (264873—501359)	77262 (62494—96594)	62783(46701—84912)	728.14 (511.76—968.68)	70.04 (56.65—87.56)	62.94 (46.82—85.13)	-7.57 (-7.70—-7.44)	-5.20 (-9.25—-0.97)	-7.45 (-7.60—-7.30)
45—49 years	176361 (126894—239165)	12592 (8745—18103)	12850 (8751—18685)	168.68 (121.36—228.74)	13.18 (9.15—18.95)	12.70 (8.65—18.47)	-8.08 (-8.20—-7.96)	-1.83 (-1.87—-1.78)	-7.94 (-8.09—-7.79)
50—54 years	458157 (331165—630460)	97619 (76884—119807)	86707 (60977—117685)	957.72 (692.26—1317.90)	80.74 (63.59—99.09)	73.24 (51.50—99.40)	-8.00 (-8.13—-7.88)	-4.76 (-7.76—-1.66)	-7.88 (-8.03—-7.73)
55—59 years	553708(400423—778868)	102901 (84577—126403)	96474 (70922—136057)	1272.94 (920.55—1790.57)	93.68 (77.00—115.08)	81.60 (59.99—115.09)	-8.72 (-8.96—-8.47)	-6.67 (-11.40—-1.69)	-8.55 (-8.80—-8.31)
60—64 years	597065 (443675—824695)	87390 (72948—106677)	85372 (63996—113371)	1684.15 (1251.48—2326.23)	119.65 (99.88—146.06)	104.84 (78.59—139.23)	-8.77 (-9.01—-8.53)	-6.39 (-11.05—-1.49)	-8.65 (-8.88—-8.41)
65—69 years	579015 (424478—782890)	123306 (101504—146813)	108796 (81406—141429)	2112.31 (1548.55—2856.08)	159.84 (131.58—190.31)	142.57 (106.67—185.33)	-8.31 (-8.54—-8.09)	-5.56 (-9.31—-1.65)	-8.27 (-8.48—-8.07)
70—74 years	444936 (318719—609076)	107019 (90808—127066)	110925 (84687—143982)	2357.07 (1688.43—3226.61)	200.48 (170.11—238.04)	181.68 (138.71—235.82)	-7.96 (-8.24—-7.68)	-4.81 (-9.09—-0.32)	-7.93 (-8.18—-7.69)
75—79 years	272272 (188795—371674)	77104 (64138—93747)	75708 (57708—102135)	2382.73 (1652.20—3252.64)	232.546 (193.441—282.741)	205.80 (156.87—277.64)	-7.48 (-7.71—-7.25)	-5.93(-10.37—-1.26	-7.46 (-7.66—-7.26)

80—84 years	104753 (73684—149443)	49199 (40639—60313)	46442 (34004—63424)	1875.90 (1319.52—2676.20)	232.35 (191.92—284.83)	206.73 (151.37—282.33)	-6.90 (-7.14—-6.67)	-5.67 (-9.03—-2.19)	-6.86 (-7.07—-6.65)
85—89 years	32246 (21661—47195)	25119 (20030—31231)	23783 (16845—33078)	1615.49 (1085.18—2364.40)	216.40 (172.56—269.05)	190.69 (135.06—265.22)	-6.73 (-6.99—-6.47)	-6.13 (-12.36—0.54)	-6.65 (-6.89—-6.41)
90—94 years	5213(3587—7426)	8200 (6168—10477)	7992 (5517—10861)	1384.39 (952.42—1971.92)	205.55 (154.62—262.61)	176.49 (121.84—239.85)	-6.54 (-6.86—-6.22)	-7.34 (-10.26—-4.32)	-6.46 (-6.75—-6.17)
95+ years	962 (655—1379)	1887 (1338—2533)	2003 (1344—2799)	1469.46 (999.77—2106.12)	182.47 (129.33—244.94)	161.34 (108.21—225.41)	-7.02 (-7.36—-6.69)	-5.97 (-8.31—-3.56)	-6.89 (-7.20—-6.58)

ASDR: age-standardized DALY rate; EAPC: estimated annual percentage change; CI: confidence interval; UI: uncertainty interval.

Supplementary Table S4. Estimated parameters of the age–period–cohort (APC) model for tuberculosis incidence, mortality, and DALYs in China, 1990–2023.

	Incidence	Deaths	DALYs
Net Drift	-6.39（-6.60，-6.19）	-10.59（-11.57，-9.59）	-9.59（-9.78，-9.40）
Age			
＜5	0.39（0.32，0.48）	0.23（0.09，0.62）	11.99（10.19，14.11）
5—9	0.20（0.16，0.25）	0.02（0.01，0.07）	1.10（0.89，1.34）
10—14	0.28（0.24，0.34）	0.02（0.01，0.06）	1.29（1.09，1.52）
15—19	0.27（0.23，0.32）	0.02（0.01，0.05）	1.28（1.10，1.48）
20—24	0.25（0.22，0.30）	0.02（0.01，0.03）	0.96（0.84，1.10）
25—29	0.22（0.19，0.25）	0.01（0.01，0.02）	0.74（0.65，0.84）
30—34	0.16（0.14，0.18）	0.01（0.01，0.02）	0.56（0.51，0.62）
35—39	0.13（0.11，0.15）	0.01（0.01，0.01）	0.43（0.39，0.48）
40—44	0.11（0.10，0.13）	0.007（0.00，0.01）	0.36（0.32，0.39）
45—49	0.03（0.03，0.04）	0.001（0，0.001）	0.05（0.04，0.06）
50—54	0.08（0.07，0.09）	0.004（0.003，0.007）	0.20（0.18，0.22）
55—59	0.08（0.07，0.09）	0.004（0.003，0.006）	0.17（0.15，0.19）
60—64	0.08（0.07，0.09）	0.004（0.003，0.006）	0.16（0.14，0.18）
65—69	0.09（0.08，0.10）	0.004（0.003，0.007）	0.15（0.13，0.17）
70—74	0.08（0.07，0.09）	0.004（0.003，0.007）	0.13（0.12，0.15）
75—79	0.07（0.06，0.09）	0.005（0.003，0.008）	0.12（0.11，0.13）
80—84	0.11（0.09，0.12）	0.005（0.003，0.009）	0.11（0.09，0.12）
85—89	0.18（0.16，0.21）	0.008（0.00，0.01）	0.13（0.11，0.14）
90—94	0.45（0.39，0.52）	0.01（0.01，0.02）	0.21（0.19，0.24）
95+	1.33（1.14，1.55）	0.03（0.02，0.05）	0.48（0.42，0.55）
Period			
1990—1994	2.21（2.08，2.36）	3.74（3.07，4.56）	3.53（3.37，3.70）
1995—1999	1.94（1.83，2.07）	2.59（2.19，3.05）	2.48（2.37，2.60）
2000—2004	1.55（1.46，1.65）	1.79（1.55，2.06）	1.73（1.65，1.81）

2005—2009	1.00（1.00,1.00）	1.00（1.00,1.00）	1.00（1.00,1.00）
2010—2014	0.72（0.68，0.77）	0.47（0.40，0.57）	0.53（0.50，0.56）
2015—2019	0.49（0.45，0.52）	0.26（0.21，0.32）	0.31（0.29，0.33）
2020—2023	0.33（0.30，0.36）	0.15（0.11，0.19）	0.19（0.18，0.21）
Cohort			
1895—1899	582.39（482.37，703.15）	6887.01（3956.29，11988.74）	4171.45（3530.90，4928.21）
1900—1904	254.34（215.06，300.79）	2566.15（1520.32，4331.40）	1629.32（1407.61，1885.96）
1905—1909	120.01（102.50，140.52）	1026.04（618.33，1702.59）	678.81（593.24，776.71）
1910—1914	71.33（61.27，83.06）	521.40（317.57，856.07）	353.88（311.69，401.78）
1915—1919	40.95（35.31，47.50）	258.78（158.78，421.76）	181.21（160.56，204.53）
1920—1924	24.34（21.06，28.14）	132.78（81.87，215.36）	96.66（86.02，108.63）
1925—1929	14.62（12.69，16.84）	68.93（42.63，111.44）	52.46（46.84，58.77）
1930—1934	9.71（8.46，11.15）	37.54（23.31，60.47）	29.83（26.71，33.32）
1935—1939	6.66（5.81，7.62）	20.61（12.82，33.11）	17.09（15.34，19.05）
1940—1944	5.03（4.40，5.74）	12.17（7.58，19.54）	10.54（9.48，11.73）
1945—1949	3.49（3.05，3.99）	6.48（3.96，10.59）	5.90（5.28，6.58）
1950—1954	1.96（1.71，2.24）	3.00（1.84，4.88）	2.82（2.53，3.13）
1955—1959	1.20（1.06，1.37）	1.50（0.92，2.45）	1.46（1.31，1.62）
1960—1964	1.00（1.00,1.00）	1.00（1.00,1.00）	1.00（1.00,1.00）
1965—1969	0.59（0.51，0.67）	0.47（0.27，0.82）	0.50（0.45，0.56）
1970—1974	0.44（0.38，0.50）	0.28（0.15，0.52）	0.31（0.28，0.35）
1975—1979	0.40（0.35，0.47）	0.21（0.11，0.42）	0.25（0.22，0.28）
1980—1984	0.40（0.34，0.46）	0.16（0.08，0.34）	0.21（0.18，0.24）
1985—1989	0.28（0.24，0.33）	0.09（0.04，0.21）	0.13（0.11，0.15）
1990—1994	0.21（0.17，0.25）	0.05（0.02，0.13）	0.08（0.07，0.10）
1995—1999	0.21（0.17，0.25）	0.037（0.01，0.11）	0.07（0.06，0.08）
2000—2004	0.20（0.17，0.25）	0.03（0.01，0.08）	0.05（0.04，0.06）
2005—2009	0.17（0.13，0.21）	0.02（0.00，0.06）	0.03（0.03，0.04）

2010—2014	0.12（0.09，0.16）	0.01（0.00，0.05）	0.02（0.01，0.02）
2015—2019	0.07（0.05，0.11）	0.004（0.00，0.05）	0.007（0.01，0.01）
2020—2023	0.06（0.03，0.10）	0.002（0.00，0.09）	0.003（0.002，0.006）
Local Drifts			
＜5	-4.61（-5.94，-3.26）	-11.09（-18.97，-2.44）	-10.25（-11.39，-9.09）
5—9	-3.78（-4.70，-2.85）	-9.66（-14.97，-4.01）	-8.60（-9.39，-7.79）
10—14	-3.32（-3.99，-2.63）	-8.96（-13.02，-4.70）	-7.58（-8.21，-6.95）
15—19	-3.00（-3.58，-2.43）	-8.36（-11.82，-4.76）	-6.66（-7.19，-6.12）
20—24	-3.00（-3.53，-2.48）	-8.00（-11.20，-4.69）	-6.22（-6.72，-5.71）
25—29	-3.51（-4.00，-3.01）	-8.17（-11.06，-5.19）	-6.48（-6.95，-6.00）
30—34	-4.41（-4.89，-3.92）	-8.82（-11.34，-6.23）	-7.32（-7.75，-6.87）
35—39	-4.60（-5.09，-4.12）	-8.88（-11.21，-6.49）	-7.69（-8.13，-7.26）
40—44	-5.43（-5.92，-4.95）	-9.51（-11.60，-7.36）	-8.55（-8.97，-8.14）
45—49	-7.03（-7.51，-6.55）	-10.92（-12.84，-8.96）	-10.14（-10.53，-9.74）
50—54	-7.93（-8.37，-7.48）	-11.83（-13.51，-10.12）	-11.14（-11.50，-10.77）
55—59	-7.93（-8.34，-7.52）	-11.93（-13.37，-10.47）	-11.26（-11.58，-10.93）
60—64	-7.68（-8.05，-7.31）	-11.76（-12.96，-10.54）	-11.08（-11.37，-10.78）
65—69	-7.78（-8.12，-7.44）	-11.87（-12.85，-10.88）	-11.14（-11.40，-10.89）
70—74	-7.62（-7.94，-7.30）	-11.58（-12.39，-10.76）	-10.81（-11.05，-10.58）
75—79	-7.78（-8.08，-7.47）	-11.47（-12.16，-10.77）	-10.69（-10.92，-10.47）
80—84	-8.55（-8.84，-8.26）	-11.81（-12.42，-11.20）	-11.08（-11.30，-10.86）
85—89	-9.32（-9.60，-9.04）	-12.26（-12.85，-11.67）	-11.58（-11.81，-11.35）
90—94	-10.21（-10.50，-9.92）	-12.97（-13.57，-12.36）	-12.33（-12.58，-12.07）
95+	-11.32（-11.65，-10.99）	-14.00（-14.69，-13.30）	-13.37（-13.67，-13.07）

Net drift, local drift, and relative risk (RR) estimates with corresponding 95% confidence intervals (CIs) are presented for each indicator. Age, period, and cohort effects were estimated using the intrinsic estimator (IE) method based on Poisson regression.
